# Therapeutic drug monitoring in children and adolescents with schizophrenia and other psychotic disorders using risperidone

**DOI:** 10.1007/s00702-022-02485-6

**Published:** 2022-03-18

**Authors:** R. Taurines, S. Fekete, A. Preuss-Wiedenhoff, A. Warnke, C. Wewetzer, P. Plener, R. Burger, M. Gerlach, M. Romanos, K. M. Egberts

**Affiliations:** 1 Department of Child and Adolescent Psychiatry, Psychosomatics and Psychotherapy, Center for Mental Health, University Hospital of Wuerzburg, Margarete-Hoeppel-Platz 1, 97080 Wuerzburg, Germany; 2 Clinic for Child and Adolescent Psychiatry and Psychotherapy, Clinics of the City Cologne GmbH, Cologne, Germany; 3 Department of Child and Adolescent Psychiatry and Psychotherapy, University Hospital of Ulm, Ulm, Germany; 4 Department of Child and Adolescent Psychiatry, Medical University of Vienna, Vienna, Austria; 5 Department of Psychiatry, Psychosomatics and Psychotherapy, Laboratory for Therapeutic Drug Monitoring, Centre for Mental Health, University Hospital of Wuerzburg, Wuerzburg, Germany

**Keywords:** Risperidone, Children, Serum concentration, Schizophrenia, Therapeutic drug monitoring, Pharmacovigilance

## Abstract

Risperidone is commonly used to treat different psychiatric disorders worldwide. Knowledge on dose–concentration relationships of risperidone treatment in children and adolescents with schizophrenia or other psychotic disorders is, however, scarce and no age-specific therapeutic ranges have been established yet. Multicenter data of a therapeutic drug monitoring service were analyzed to evaluate the relationship between risperidone dose and serum concentration of the active moiety (risperidone (RIS) plus its main metabolite 9-hydroxyrisperidone (9-OH-RIS)) in children and adolescents with psychotic disorders. Patient characteristics, doses, serum concentrations and therapeutic outcomes were assessed by standardized measures. The study also aimed to evaluate whether the therapeutic reference range for adults (20–60 ng/ml) is applicable for minors. In the 64 patients (aged 11–18 years) included, a positive correlation between daily dose and the active moiety (RIS_am_) concentration was found (*r*_s_ = 0.49, *p* = 0.001) with variation in dose explaining 24% (*r*_s_^2^ = 0.240) of the variability in serum concentrations. While the RIS_am_ concentration showed no difference, RIS as well 9-OH-RIS concentrations and the parent to metabolite ratio varied significantly in patients with co-medication of a CYP2D6 inhibitor. Patients with extrapyramidal symptoms (EPS) had on average higher RISam concentrations than patients without (*p* = 0.05). Considering EPS, the upper threshold of the therapeutic range of RIS_am_ was determined to be 33 ng/ml. A rough estimation method also indicated a possibly decreased lower limit of the preliminary therapeutic range in minors compared to adults. These preliminary data may contribute to the definition of a therapeutic window in children and adolescents with schizophrenic disorders treated with risperidone. TDM is recommended in this vulnerable population to prevent concentration-related adverse drug reactions.

## Introduction

Risperidone is commonly used worldwide to treat a variety of psychiatric symptoms and disorders in children and adolescents (Halfdanarson et al. 2017). Risperidone has FDA approval to treat schizophrenia in patients from 13 to 17 years as well as manic episodes in patients with bipolar disorder from 10 to 17 years. Furthermore, it is licensed in most countries for the short-term treatment of aggressive behavior in children and adolescents in the age of 5 years or older with mental retardation or irritability in children with autism spectrum disorder (ASD). However, in clinical practice, risperidone is often used off-label for a much broader range of mental disorders in young patients, such as tic disorders (Kim et al. 2005; Ghanizadeh and Haghighi 2014) or impulsive aggressive behavior associated with attention deficit hyperactivity disorder (Gadow et al. 2016).

While various randomized controlled trials and meta-analyses have confirmed the effectiveness/efficacy of risperidone (Lee et al. 2018) for different clinical indications, safety concerns remain given its adverse drug reactions (ADRs) profile. Especially weight gain and sedation seem to be more pronounced in youth (Safer 2004; Liu et al. 2019), but also extrapyramidal symptoms (EPS), hyperprolactinemia and impaired glucose tolerance are common ADRs that can lead to serious long-term health risks (Solmi et al. 2020). As some of these ADRs seem dose-dependent, optimal dose finding with the lowest effective dose is demanded. However, dosing in the age group of minors is difficult, due to age and developmental dependent differences in pharmacokinetics/pharmacodynamics (Egberts et al. 2011) and limited dosing evidence for off-label indications. Due to these benefit/risk concerns a close monitoring of the patient is important.

Therapeutic drug monitoring (TDM) provides a valid method for individual dose titration and careful monitoring and is strongly recommended by guidelines for adults treated with risperidone (Hiemke et al. 2018; Schoretsanitis et al. 2020). The parent component risperidone (RIS) is mainly metabolized by CYP2D6 into 9-hydroxyrisperidone (9-OH-RIS). The pharmacological characteristics and therapeutic effects of 9-OH-RIS are considered comparable to those of the parent compound (Heykants et al. 1994; Nazirizadeh et al. 2010). For this reason, TDM guidelines advise to determine serum concentrations of RIS and 9-OH-RIS and the sum of both, which is referred to as the *active moiety* (RIS_am_) concentration in this paper*.* For adult patients with schizophrenia or bipolar disorder concentrations in the range of 20–60 ng/mL of the active moiety are recommended. Serum concentrations > 40 ng/mL should be targeted only in patients with insufficient or absence of therapeutic response to avoid neurological adverse reactions (Hiemke et al. 2018).

In children and adolescents, however, the relationships between risperidone dose and blood concentrations have not been clarified yet and age-specific recommended reference ranges of blood concentrations have not been defined for the different age or diagnostic groups. The few existing pharmacokinetic studies in pediatric patients all revealed linearity between daily dose and blood concentrations of the active moiety (Klampfl et al. 2010; Calarge and Miller del 2011; Fekete et al. 2021). In a population-based study, risperidone serum concentrations were comparable between children and adolescents and in line with results from adult patients (Thyssen et al. 2010). In a retrospective naturalistic study, however, lower dose-corrected serum concentrations of RIS_am_ were found in minors compared to adults possibly due to a higher renal clearance (Fekete et al. 2021). Other studies identified age and/or gender as well as body fat as potential modifying factors of risperidone concentrations (Aichhorn et al. 2007a; Calarge and Miller del 2011).

Several studies reported significant correlations between RIS or 9-OH-RIS concentrations and hyperprolactinemia (Troost et al. 2007; Duval et al. 2008; Migliardi et al. 2009; Roke et al. 2012; Ngamsamut et al. 2016) in youth, in contrast to another study that found no correlation between RIS_am_ concentrations and prolactin level increase in pediatric patients (Gagliano et al. 2004). A recent prospective cohort study in children and adolescents with ASD revealed that higher RIS_am_ trough concentrations predict higher prolactin levels, but also higher body mass index scores, more sedation and, interestingly, more effectiveness reducing irritability, indicating that a therapeutic window seems to exist for this indication (Kloosterboer et al. 2021). However, clinical studies assessing the relationships between psychotropic drug concentrations and clinical outcomes in children and adolescents are rare and characterized by methodological limitations (Kloosterboer et al. 2020).

The primary aim of this study was to assess the relationship between daily dose and serum concentration in children and adolescents treated with risperidone due to a schizophrenic psychosis using data from a routine TDM service. As a secondary aim, the influence of several patients’ and treatment characteristics on the serum concentrations was investigated. Furthermore, the aim was to explore whether the recommended range for blood concentrations of the active moiety in adults seems also valid and applicable for children and adolescents with schizophrenia.

## Subjects and methods

### Setting and study population

Patient data and blood samples were collected from three university hospital departments (Ulm, Wuerzburg, Vienna) and two departments of child and adolescent psychiatry (Ravensburg, Cologne Holweide) in Germany and Austria between 2006 and 2018 (Table 1). All participating hospitals are members of the competence network for TDM in child and adolescent psychiatry (www.tdm-kjp.com, Mehler-Wex et al. 2009) and used the routine TDM service of the service of the Center of Mental Health of the University Hospital Wuerzburg. Within this TDM service, serum concentrations of psychotropic drugs are measured and demographic, psychiatric and outcome data collected in a standardized way.

**Table 1:** Characteristics of study population (*N* = 64).

Clinical Center, *N* = 64, *N *(%)	
Cologne	28 (43.8)
Wuerzburg	27 (42.2)
Weissenau	4 (6.3)
Ulm	4 (6.3)
Vienna	1 (1.6)
Gender, *N* = 64, *N* (%)	
Male	45 (70.3)
Female	19 (29.7)
Age (years), *N* = 64, mean ± SD, range	15.6 ± 1.7, 11–18
Children < 12 years	3 (4.7)
Adolescents ≥ 12 years	61 (95.3)
Weight (kg), *N* = 61, mean ± SD, range	67.0 ± 15.7, 30–108
Height (cm), *N* = 60, mean ± SD, range	170.0 ± 12.0, 142–193
BMI (kg/m^2^), *N* = 58, mean ± SD, range	23.4 ± 4.7, 14.2–36.4
Smoking, *N* = 61, *N* (%)	10 (16.4)
Most common ICD diagnosis, *N* = 64, *N* (%), multiple entries	
F 1x.5 drug induced psychosis	4 (6.3)
F 20.x schizophrenic disorders	45 (70.4)
schizotypal disorder	4 (6.3)
F 23.x acute transient psychotic disorders	5 (7.8)
F 25.x schizoaffective disorders	5 (7.8)
F 28.0 other non-organic psychotic disorders	1 (1.6)
Severity of illness (CGI-S), *N* = 60, *N *(%)	
Not at all ill	1 (1.7)
Borderline mentally ill	1 (1.7)
Mildly ill	2 (3.3)
Moderately ill	9 (15.0)
Markedly ill	30 (50.0)
Severely ill	17 (28.3)
Extremely ill	0 (0.0)
Risperidone monotherapy, *N* = 63, *N* (%)	13 (20.6)
Co-medication, *N* = 63, *N* (%)	50 (79.4)
Psychiatric co-medication, *N* = 63, *N* (%), multiple entries.	
Antipsychotics (*aripiprazole*, *haloperidol*, *clozapine*, *quetiapine*, *olanzapine*, *melperon*, *pipamperone*, *chlorprothixen*, *levomepromacin*, *promethazine*, *perazine*, *amisulprid*)	30 (47.6)
Biperiden	20 (31.7)
Tranquilizer	15 (23.8)
Antidepressants (*escitalopram*, *fluoxetine*, *fluvoxamine*, *mirtazapine*)	7 (11.1)
Potential CYP2D6-inhibitors^a^ (*fluoxetine n *= 4,* citalopram*, *fluvoxamine*)	6 (9.5)
Mood stabilizers/anticonvulsants	3 (4.7)
Beta blockers	3 (4.7)
Stimulants	1 (1.6)
Other	9 (14.3)
Clinical outcome (CGI-I), *N* = 60, *N* (%)	
(Very) much better^(1)^	20 (33.3)
Moderately better^(2)^	23 (38.3)
Unchanged/slightly worse	12 (20.0)
Much worse	4 (6.7)
Not assessable	1 (1.7)
“Responder”^(1+2)^	43 (71.6)
Adverse Drug reactions (UKU), *N* = 63, *N* (%)	
Documented side effects	40 (63.5)
Severity of ADRs (UKU), *N* = 40, *N *(%)	
Mild	23 (57.5)
Moderate	17 (42.5)
Severe	0 (0.0)

*CGI-I  * Clinical Global Impression Scale–Improvement, *CGI-S* Clinical Global Impression Scale–Severity, *UKU*  Udvalg for Kliniske Undersogelser-Side Effect Rating Scale, *N* = number of patients, *SD* = standard deviation

^a^According to: https://www.fda.gov/drugs/drug-interactions-labeling/drug-development-and-drug-interactions-table-substrates-inhibitors-and-inducers.

**Table 2:** Risperidone daily doses and serum concentrations in different subsample

Patients (N)	Risperidone daily dose Mean ± SD median, (IQR) (mg/day)	Concentration RIS_am_ (active moiety) Mean ± SD median, (IQR) (ng/ml)	Concentration RIS Mean ± SD median, (IQR) (ng/ml)	Concentration 9-OH-RIS Mean ± SD median, (IQR) (ng/ml)	Correlation daily dose RIS_am_ concentration (*r*_s,_,*p*)	Group differences in RIS_am_ concentrations (*p*)
All (64)	3.9 ± 1.9 4.0 (2.0–5.4)	32.2 ± 22.1 27.5 (17.0–43.8)	8.6 ± 11.1, 3.5 (2.0–12.0)	23.7 ± 16.8, 20.5 (12.2–30.0)	0.49, <0.001	
Boys (45)	4.0 ± 2.0 4.0 (2.0–5.5)	23.1 ± 21.1 28.0 (17.0–43.5)	9.2 ± 11.1 4.0 (2.0–15.5)	22.6 ± 15.8 20.0 (11.5–30.0)	0.59, <0.001	0.97
Girls (19)	3.6 ± 1.5 3.0 (2.5–4.5)	33.4 ± 24.8 26.0 (16.0–44.0)	7.1 ± 11.2 3.0 (2.0–9.0)	26.3 ± 19.3 23.0 (13.0–31.0)	0.27, 0.26	
Mono-therapy (13)	4.2 ± 1.5 4.5 (3.0–5.8)	22.2 ± 8.4 24.0 (17.0–29.0)	3.8 ± 4.3 3.0 (1.54.0)	18.5 ± 8.5 20.0 (10.6–26.0)	0.37, 0.21	0.10
Co-medication (50)	3.7 ± 1.9 3.5 (2.0–5.0)	34.9 ± 24.0 30.0 (15.8–44.0)	10.0 ± 12.0 4.0 (2.0–13.3)	25.0 ± 18.4 21.0 (12.8–33.0)	0.56, <0.001	
Responders (43)	3.8 ± 2.0 4.0 (2.0–5.0)	31.9 ± 23.3 26.0 (16.0–43.0)	7.8 ± 9.9 3.0 (3.0–13.0)	24.4 ± 17.9 20.5 (12.0–31.0)	0.57 <0.001	
Non-Responders (16)	4.0 ± 1.8 4.5 (3.0–5.5)	33.8 ± 21.3 29.0 (17.0–44.0)	9.7 ± 14.1 3.5 (2.0–9.0)	24.2 ± 17.1 20.5 (11.8–29.5)	0.19 0.49	0.77

RIS_am_ (active moiety) = sum concentration of risperidone (RIS) and its active metabolite 9-OH-risperidone (9-OH-RIS).

All patients—as part of routine patient care—received a physical–neurological and psychiatric examination, assessment of vital signs, body size, body weight, and laboratory analyses for hepatic and renal function. In addition, further patient characteristics (gender, age, diagnosis, comorbidity, nicotine use) and clinical data of drug treatment (e.g., dosage of risperidone, type and dosage of possible psychiatric co-medications) were determined. Furthermore, the date, reason for TDM analysis (e.g., ‘dose adjustment’ or ‘compliance control’) and the symptoms intended to treat with the medication (e.g., ‘positive symptoms’) were systematically assessed.

All patients up to the age of 18 years who were administered risperidone for the treatment of schizophrenic disorders (ICD-10 F2; mostly F20.x) or a drug induced psychotic disorder (ICD-10 F12.5, F19.5) independently of the setting of treatment (inpatient, outpatient, day-unit) were included. Patients were excluded from the study if steady-state conditions for blood taking were not fulfilled or relevant data were missing (e.g., daily dose, relevant patient information). Patients with drug induced psychotic disorder were excluded in case of ongoing consumption of illegal drugs.

The study was approved by the local ethic committee (study number 27/04) and carried out according to the Declaration of Helsinki. As the investigation of serum concentrations was part of the clinical routine of blood tests there was no necessity for written informed consent.

### Measurement of risperidone serum concentrations

Analyses of RIS/ 9-OH-RIS serum concentrations were performed according to the consensus guidelines of TDM in Psychiatry of the AGNP (Arbeitsgemeinschaft für Neuropsychopharmakologie und Pharmakopsychiatrie; German society for neuropsychopharmacology and pharmacopsychiatry (Hiemke et al. 2018). In steady-state conditions, blood withdrawal from cubital veins was performed in 7.5 mL monovettes without anticoagulants and additives as trough value before the first daily intake of risperidone. The elimination half-life of RIS is 2–4 h, of its metabolite 9-OH-RIS 17–23 h, and of the active moiety 20 h (Mannens et al. 1993; Borison et al. 1994). Steady plasma concentrations are reached in poor metabolizers by days 5–6, in extensive metabolizers by day 1 (Chopko and Lindsley 2018). Date and time of blood withdrawal were noted. The blood was centrifuged at 1800 g for 10 min and analyzed immediately (samples from Wuerzburg) or within a few days after postage to the TDM laboratory in Wuerzburg.

Serum concentrations of RIS and 9-OH-RIS were analyzed by an automated column-switching method coupled to an isocratic high-performance liquid chromatography (HPLC) system and a variable ultraviolet detector as described in detail elsewhere (Klampfl et al. 2010). The intra-assay coefficients of variation determined from 10 analyses of both analytes (20 and 80 ng/mL) were in general less than 1%. The inter-assay variability for both analytes was in general less than 2%. The method was linear in a range of 4–200 ng/mL (RIS *r*^2^ = 0.99, 9-OH-RIS *r*^2^ = 0.99), and the lower limit of quantification was 3 ng/mL for both compounds. Chemicals and solvents with level of purity and RIS and 9-OH-RIS for calibration and controls were purchased commercially from Sigma-Aldrich, Munich, Germany. For patients with more than one concentration determination, the chronologically last available measurement was selected for this study.

### Assessment of therapeutic outcomes

To assess the severity of psychopathology and the change of symptomatology, at the time of blood withdrawal, the Clinical Global Impression Scale was used (severity: CGI-S; improvement: CGI-I) and the change (improvement) therein (CGI-I) as a measure for effectiveness (Guy 1976). The following categories were applied adapting the CGI manual: 1 = (very) much better, 2 = moderately better, 3 = unchanged/ slightly worse, 4 = much worse, 0 = treatment effect not assessable. Patients with a CGI-I score of 1, 2 were defined as responders to drug treatment. The nature and severity of ADRs at the time period before blood taking were assessed using the Udvalg for Kliniske Undersogelser Side Effect Rating Scale (Lingjaerde et al. 1987) with the following categorization: 0 = no side effects; 1 = mild, 2 = moderate and 3 = severe side effects.

### Data analysis

Statistical analyses were performed with the software SPSS, version 26. Means, medians and interquartile ranges (IQRs) were calculated for descriptive analyses. The Kolmogorov–Smirnoff test was used to evaluate variables for Gaussian distribution. The Spearman rank correlation coefficient (*r*_s_) was applied for not Gaussian distributed variables (serum concentrations), the Pearson coefficient (*r*_p_) for Gaussian distributed variables. Group differences were analyzed by independent *t* test and Mann–Whitney *U* test. Multiple linear regression analysis was used to determine influencing factors on RIS, 9-OH-RIS and RIS_am_ concentrations, e.g., sex, comedication, body weight, body mass index and cigarette smoking. A receiver-operating curve (ROC analysis) was performed to determine the upper limit of an age-specific therapeutic reference range, analyzing the RIS_am_ concentrations of patients without and with ADRs (EPS) to identify a cutoff value that separates patients with a high probability of such ADRs from those with a low probability. Statistical significance was defined as *p* ≤ 0.05. All values are presented as mean ± SD or as median and IQR whatever appropriate. The data set generated and analyzed during the current study is available from the corresponding author on reasonable request.

## Results

### Study population

The study population comprised 64 patients (70.3% male) with a mean (SD, range) age of 15.6 (1.7, 11–18) years, of whom three (4.7%) were younger than 13 years (Table 1). The vast majority (93.7%) had a diagnosis of a schizophrenic disorder (ICD-10 F2), 6.3% were diagnosed with psychotic symptoms in the context of consumption of illegal drugs (ICD-10 F1x.5). Almost 80% of the patients received one or more concomitant psychotropic medications, most commonly other antipsychotics. The severity of symptomatology was in most patients classified as ‘markedly ill’ (50.0%) or ‘severely ill’ (28.3%).

#### RIS/9-OH-RIS and active moiety concentrations in relation to risperidone doses and other covariates

The patients were treated with an average of 3.9 (SD 1.9, range 1–8) mg risperidone daily (Table 2). The mean body weight-adjusted dose was 0.02 mg/kg (SD 0.01, range 0.009–0.043). The daily dose did not differ in the subgroups classified according to gender (*p* = 0.46) or mode of pharmacotherapy (monotherapy versus co-medication) (*p* = 0.35). The mean dose-corrected RIS_am_ concentration (C/D) was 9.2 (ng/ml) / (mg/day) (SD 6.2, range 2.3–29.3). There was no statistically significant difference in the dose-corrected RIS_am_ concentrations between boys 8.2 (ng/ml)/(mg/d) and girls 10.6 (ng/ml)/(mg/day) (*p* = 0.24).

**Fig. 1:**
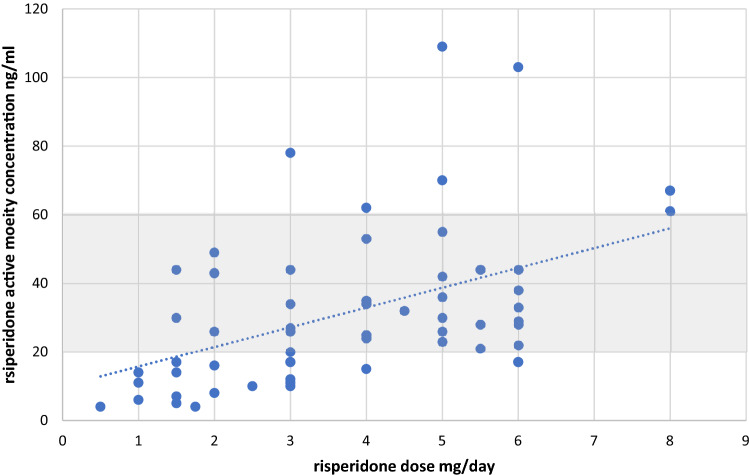
Relationship between risperidone dose per day and the RIS_am_ (active moiety = RIS plus 9-OH-RIS) steady-state trough serum concentrations for *n* = 64 patients. The recommended therapeutic range of RIS_am_ concentrations (20–60 ng/ml) in adults is highlighted

In 22 (34%) individuals, the RIS concentration was below, whereas the concentration of 9-OH-RIS was above the lower limit of quantification of the assay in all patients. The mean (SD) RIS_am_ concentration (*n* = 64) was 32.2 ng/ml (22.1). A large inter-patient variability of RIS_am_ concentrations was shown (IQR 17.0–43.8 ng/ml). Table 2 shows the measured serum concentrations in the total population and the different subsamples. In the whole sample a positive correlation between daily doses and RIS_am_ concentrations was found (*r*_s_ = 0.49, *p* = 0.001), with the variation in dose explaining 24% (*r*_s_^2^ = 0.24) of the variability in serum concentrations (Fig. 1). Multiple linear regression analysis confirmed that sex (*p* = 0.79), body weight (*p* = 0.92), body mass index (*p* = 0.47) and the use of any psychotropic comedication versus monotherapy (0.07) had no significant influence on the RIS_am_ concentration. In contrast, cigarette consumption significantly influenced serum concentrations (*p* = 0.045) since RIS concentrations, 9-OH-RIS as well as RIS_am_ concentrations were each about 45% lower in smokers.

We identified six patients using a CYP2D6 inhibitor as concomitant medication, mainly fluoxetine (Table 2). Whereas there was no difference in the RIS_am_ concentration (*p* = 0.87), both the RIS (*p* = 0.004) and 9-OH-RIS levels (*p* = 0.026) as well as the RIS/9-OH-RIS ratio (*p* = 0.003) were significantly different between patients without and with a CYP2D6-inhibiting concomitant medication (more parent substance and less metabolite in patients using a CYP2D6 inhibitor).

#### Clinical positive and negative effects of risperidone treatment

Using CGI-I, 33.3% (*n* = 20) of the children and adolescents were rated as ‘(very) much better’ and 38.3% (*n* = 23) as ‘moderately better’. These 43 (71.6%) patients (were classified as responders. 26.7% of the patients were classified as non-responders as their state was rated as ‘unchanged/slightly worse’ (20%) or as ‘(much) worse’ (6.7%). Girls and boys were equally likely to be responders (*p* = 0.80). There was no significant difference of RIS_am_ concentrations in responders compared to non-responders (*p* = 0.77) (Table 2).

One or more ADRs were documented in the majority (63.5%) of the patients, with a non-significant higher frequency in girls (72.2%) than boys (60.0%) (*p* = 0.36). There also was no significant difference in the occurrence of ADRs in patients with and without concomitant psychotropic medication (*p* = 0.44). In the group of patients with ADRs and a rating of the treating child psychiatrist on severity (*n* = 32), 56.3% of ADRs were rated as ‘mild’, 43.7% as ‘moderate’. ‘Severe’ ADRs did not occur in any of the patients. Sedation/drowsiness (*n* = 18; 21.1% of all patients) and EPS (*n* = 13; 19.1% of all patients) were the most frequently reported ADRs. Other ADRs comprised an inner feeling of tension and agitation, hypersalivation, micturition and accommodation problems, gastrointestinal disturbances and cardiovascular disturbances. Prolactine was not measured in our study. Within a mean (SD) observation period from 156 ± 328 days (range 8–1190) in a small subgroup of 14 patients, an average weight gain (mean, SD) of 3.2 ± 4.6 kg (range: −1 to 14) was recorded. RIS_am_ concentrations did not differ in patients with and without any kind of ADRs (*p* = 0.60). However, patients with EPS (*n* = 13) had significantly higher (*p* = 0.05) mean RIS_am_ concentration (42.9 ng/ml) than patients without EPS (29.5 ng/ml), the same was true for RIS and 9-OH-RIS concentrations.

### Determination of a preliminary therapeutic reference range of risperidone in children and adolescents with schizophrenic disorders

The comparison of RIS_am_ concentrations in our pediatric sample with the currently recommended therapeutic levels of the active moiety for adult patients with schizophrenia (20–60 ng/ml) revealed that 20 (31.3%) of all measured RIS_am_ concentrations were under the recommended therapeutic level for adults, and 8 (12.5%) were above. 36 (56.3%) of the RIS_am_ concentrations were within the therapeutic window—of which 25 (39.1%) were in the range between 20 and 40 ng/ml. No measured concentration reached the so-called laboratory alert level of 120 ng/ml, a threshold that obliges the laboratory to feedback immediately to the prescribing physician.

As patients with EPS had higher RIS_am_ concentrations than patients without EPS, we conducted a ROC analysis to identify the upper threshold of a possible age-specific therapeutic range. The area under the curve (AUC) was 0.63. The best distinction between sensitivity and specificity was found at a RIS_am_ concentration of 33 ng/ml indicating this cut off-value as the upper limit.

As long as valid data on a therapeutic reference range do not exist, a method described by the consensus guidelines for TDM in neuropsychopharmacology (Hiemke et al. 2018, for details see the discussion) can be applied for a rough estimation of a therapeutic window considering the range between the arithmetic mean ± one standard deviation of drug concentrations in the blood of responders to the drug therapy. We used this approach to estimate the lower limit of the therapeutic window of risperidone for children and adolescents with schizophrenic disorders. The mean (SD) RIS_am_ concentration of all responders was 31.9 (± 23.3) ng/ml leading to a suggested lower limit of about 9 (8.6) ng/ml.

## Discussion

In this observational study in children and adolescents with schizophrenia, a positive linear relationship between daily dose and the RIS_am_ concentration was found. Smoking reduced RIS and 9-OH-RIS concentrations by about 45%. Concomitant medication of a CYP2D6 inhibitor had significant influence on the RIS concentrations (parent compound increased) as well as the metabolite 9-OH-RIS (reduced). Patients with EPS had higher RIS as well as 9-OH-RIS concentrations than patients without. More than a half of the pediatric patients had a RIS_am_ concentration within the recommended therapeutic range for adults with schizophrenia. Using an estimation method for the determination of a preliminary therapeutic window, our data point to a similar upper limit, but a decreased lower limit of a therapeutic range for children and adolescents with schizophrenia treated with risperidone.

### Risperidone concentrations in relation to risperidone doses

In line with former studies on pharmacokinetics of risperidone in children and adolescents (Klampfl et al. 2010; Calarge and Miller del 2011; Fekete et al. 2021), a linear correlation between dose and blood concentration was observed. Our finding also is in line with a correlation between daily dose and risperidone blood concentrations seen in adult patients (He and Richardson 1995; Bowskill et al. 2012). As in previous pediatric TDM studies (Aman et al. 2007; Klampfl et al. 2010), and studies on adults (Aravagiri et al. 1998, 2003; Zhou et al. 2006; Castberg et al. 2017), concentrations in our pediatric sample were widely distributed and dosage accounted for only 24% of the variability in RIS_am_ serum concentrations. We found no significant influence of sex, body weight, body mass index on serum concentrations, neither an influence of age in our quite age-homogeneous sample (age 11–18 years). In the literature, an effect of old age on risperidone concentrations was observed with dose-adjusted concentrations about twice as high in the age of 80 compared to the age of 40 (Castberg et al. 2017). Heterogeneous results on sex differences were reported, as some studies found higher serum concentrations in females (e.g.,Aichhorn et al. 2007b; Castberg et al. 2017) and some in boys/males (Calarge and Miller del 2011). Others studies found no influence of sex (Thyssen et al. 2010; Pozzi et al. 2016; Kloosterboer et al. 2021), especially when the sample mainly consisted of boys like in our study, which is in line with the reported ratios of boys to girls of about 2.5:1 in schizophrenic patients with onset during childhood and adolescence (Russell et al. 1989). The differing results on sex might be confounded by age effects on renal clearance and maturation of the CYP system, duration of antipsychotic treatment as well as concomitant CYP2D6 inhibiting medication that was not controlled for in some studies. A higher renal clearance of 9-OH-RIS in minors might also be the reason for lower metabolite to parent-ratios (MPRs) of risperidone found in children/adolescents compared to adults (Fekete et al. 2021). In contrast to our results, other studies reported that a higher BMI correlates with higher 9-OH-RIS partly due a to altered CYP2D6 or CYP3A4 activity in obese patients (Paulzen et al. 2016).

In our sample cigarette consumption significantly influenced blood levels since RIS concentrations, 9-OH-RIS as well as RIS_am_ concentrations were each about 45% lower in smokers. The influence of cigarette smoking is vastly discussed in the literature with a well know effect of CYP1A2 induction by polycyclic aromatic hydrocarbons in cigarette smoke. Apart from the induction of CYP1A2 activity, Schoretsanitis et al. (2017) suggest from their retrospective TDM database study on nearly 700 patients, that smoking might exert an effect on additional CYP isoenzymes as well, most likely via CYP3A4. However, there is only one study which investigated the influence of polymorphisms in other cytochrome P450 systems than CYP2D6 for risperidone in children and adolescents, but did not find any significant effects of these genes (CYP3A4, CYP3A, P-glycoprotein) on risperidone pharmacokinetics (Kloosterboer et al. 2021).

While there was no difference in the RIS_am_ concentration under comedication with potential CYP2D6 inhibitors in our small subsample (*n* = 6), we identified that concomitant CYP2D6 inhibitors modified the MPR with an increased parent compound and reduced metabolite concentration. CYP2D6-inhibiting co-medications, have been demonstrated to increase the concentration–dose ratio of RIS, and coadministration of the potent inhibitor fluoxetine increased the RIS_am_ concentration by 50–75% (Chopko and Lindsley 2018); a finding in line with former results in adult patients (Berecz et al. 2002). In the literature a certain tissue distribution was found with a higher brain-to-plasma concentration ratio for RIS parent compound than for its active metabolite compared to other tissues, such as kidneys and liver (van Beijsterveldt et al. 1994; Aravagiri et al. 1998; Calarge and Miller del 2011). Therefore, co-administered CYP2D6 inhibitors during risperidone therapy could alter the serum concentrations of RIS and 9-OH-RIS and by the differing tissue distribution modify disposition in the brain, with a clinically significant implication on efficacy and tolerability. As clinical trials on polypharmacy to risperidone are scare, although polypharmacy is increasingly prevalent in daily clinical practice in youth (Safer et al. 2003; dosReis et al. 2005; Zito et al. 2008; Vloet et al. 2019), our real world data add to current knowledge.

Our data are only partially comparable with previous studies on risperidone blood levels in children and adolescents, as these studies have addressed patients with different diagnoses in mixed samples (Pozzi et al. 2016; Fekete et al. 2021) or other specific diagnostic subgroups, e.g., patients with behavioral disorders or ASD (Gagliano et al. 2004; Klampfl et al. 2010; Calarge and Miller del 2011; Kloosterboer et al. 2021). The mean daily risperidone doses, therefore, were comparably higher in our sample, consistent with the published literature to treat early onset schizophrenia (Armenteros and Davies 2006) and in contrast to the recommended lower dosages to treat behavioral problems (Ipser and Stein 2007).

### Clinical outcome

Risperidone treatment was estimated as effective in most of the patients, as the symptoms of one third of the patients of the children and adolescents were rated as ‘(very) much better’ and more than 70.0% of the patients showed at least some benefit under the pharmacotherapy (‘moderately better’). ADRs were very common and documented for the majority of children and adolescents (63.5%). However, all ADRs were rated as ‘mild’ and ‘moderate’. ‘Severe’ ADRs did not occur in any of the patients. From the literature it is known, that ADRs correlate with RIS_am_ concentrations (Hiemke et al. 2018). In addition, elevated levels of dose-adjusted plasma 9-OH-RIS concentration were found to be associated with higher rates of ADR (Schoretsanitis et al. 2016). In line with literature in adult patients with schizophrenia (Spina et al. 2001), also in our sample, patients with EPS had significantly higher mean RIS, 9-OH-RIS and RIS_am_ concentrations than patients without. In the literature, in some studies a ratio > 1 of RIS/9-OH-RIS reflecting diminished CYP2D6 activity was associated with higher rates of ADR (Leon et al. 2007). In our sample, a subgroup of only 8 patients had an inverted ratio and no such correlation could be found.

### Proposal of a preliminarily therapeutic range for children and adolescents

In all age groups, TDM of patients under risperidone treatment is recommended for personalized pharmacotherapy in clinical routine (for dose titration as well as special indications like assessment of medication adherence), as some ADRs correlate with drug concentrations and TDM increases the probability of response. For adults with schizophrenia, the therapeutic reference range of the RIS_am_ concentration is defined as 20–60 ng/ml according to results of PET studies and studies with therapeutically effective doses (Hiemke et al. 2018).

In children and adolescents treated with risperidone, no controlled TDM studies with fixed dose designs nor PET studies are available. A previous prospective clinical study indicates that a therapeutic window for risperidone in children and adolescents with ASD exists, as the RIS_am_ concentrations predicted both side effects and response (Kloosterboer et al. 2021). In addition, for children and adolescents treated with low doses of risperidone due to impulsive–aggressive behavior, a therapeutic window (8–26 ng/ml) was suggested (Klampfl et al. 2010). To our knowledge, valid data on a therapeutic reference range of risperidone in patients with (early onset) schizophrenia do not exist. A comparison of our data with the recommended therapeutic levels for adults revealed that more than half of the pediatric patients of our sample had a RIS_am_ concentration within the recommended therapeutic range for adults (56.3%) and 31.3% had a lower concentration.

Using a ROC analysis separating patients with EPS side effects from those without, an upper limit of a possible age-specific therapeutic range of RIS_am_ concentrations in children and adolescents with schizophrenic disorders of 33 ng/ml could be identified. Because of the limitations of our naturalistic study design it was not impossible to calculate the lower threshold level of the therapeutic range by a ROC analysis. Therefore, we referred to the method described in the consensus guidelines for TDM in neuropsychopharmacology (Hiemke et al. 2018) and used the arithmetic mean minus one standard deviation of the drug concentrations in the blood of responders to estimate the lower limit. Altogether, our findings suggest a therapeutic reference range of RIS_am_ from 9 to 33 ng/ml in children and adolescents, which is significantly lower than the range for adults (20–60 ng/ml) with schizophrenic disorders, but still higher than the range for children and adolescents treated with risperidone due to behavioral problems. Of course, this does not mean that all pediatric patients with schizophrenia already respond under lower RIS_am_ concentrations. However, dose has to be titrated very carefully, and we can underline for minors—like stated for adults in the TDM consensus guidelines for blood levels > 40 ng/mL (Hiemke et al. 2018)—that RIS_am_ > 33 ng/ml should be targeted only in cases of insufficient or absence of therapeutic response to avoid neurological adverse reactions. For the use of risperidone in other than the main indication, therapeutic concentration ranges have to be evaluated for the different age groups in further studies.

### Limitations and strengths of the study

The findings of the present study must be interpreted in the context of several limitations. First, the influence of age and pubertal stage could not be determined, because only three children younger than 13 years were involved and the information on puberty was not recorded. Second, no genotyping was performed for the cytochrome P450 enzymes (CYP2D6, CYP3A4 and P-glycoprotein), that could influence pharmacokinetics. Third, the sample size was limited and especially the different subgroups of patients too small to identify variables with small effect sizes. Fourth, our study goes along with the typical limitations of an observational–naturalistic design including non-standardization of dose regimes and length of drug treatment before TDM assessment. Finally, concentration–effect relationships are very difficult to determine within naturalistic studies (Hiemke 2019) as placebo-responders and patients with side effects, who are likely to receive lower dosages, are not excluded from analysis as well as non-responders, who are likely to receive high dosages. To define a therapeutic window, further studies with more controlled study designs are necessary with higher case numbers and fixed dose regimens, fixed points of response assessment and the use of more specific clinical instruments to define clinical response for the different diagnostic entities or to investigate dose dependent side effects. Therefore, to complement naturalistic studies, there is the urgent need for more standardized investigations with larger sample sizes and controlled clinical designs. To learn more about the benefit–risk profile of psychotropic drug use in daily clinical practice a multicenter pharmacovigilance study (‘TDM-VIGIL’) was funded by the German Federal Institute for Drugs and Medical Devices (BfArM), Bonn, in collaboration with the ‘Competence Network on TDM in Child and Adolescent Psychiatry’’ (www.tdm-kjp.com). Using a modern internet-based patient registry, epidemiological and outcome data were assessed in a standardized way, including the results of standardized analysis of serum concentrations (Egberts et al. 2015, 2022).

As a strength, observational TDM studies like the present allow to gain data on dose–concentration relationships in ‘real world patients’, who are characterized by a variety of individual clinical characteristics and concomitant medications. For example, in clinical trials patients who use multiple psychotropic medications, are usually excluded. Only about 20% of the young patients in our sample were prescribed risperidone monotherapy, all others received at least one concomitant psychotropic drug in addition to risperidone, although monotherapy with antipsychotics is recommended as the first-line treatment for schizophrenia in most clinical guidelines (e.g.,Lehman et al. 2004; Miller et al. 2004 German Association for Psychiatry, Psychotherapy and Psychosomatics 2019), while polypharmacy with psychotropic agents in the treatment of schizophrenia is common in clinical practice (Hashimoto et al. 2021). Nearly half of the patients received two or more antipsychotics (antipsychotic polypharmacy), which is common in the treatment of schizophrenic disorders for the management of refractory psychotic symptoms (Correll and Gallego 2012). The observed considerable inter-individual differences in serum concentrations could partly be influenced by 2D6 activity either by genetic variation or by the use of 2D6 inhibiting comedication. There are more than 70 variants identified for CYP2D6 (Zhou 2009a, b) and an effect of CYP2D6 variants on the clearance of RIS metabolites is proven: poor metabolizers achieved RIS_am_ concentrations up to 3.3-fold (intermediate 1.6-fold) higher at the same doses compared to extensive metabolizers (Riedel et al. 2005; Locatelli et al. 2010; Hendset et al. 2014). In the future, genotyping of CYP2D6 as part of the clinical routine—additionally to TDM—might help for a more personalized pharmacotherapy with individual dose optimization.

### Conclusion

Our study described the distribution of risperidone serum concentrations of pediatric patients with schizophrenia in a real life-setting, significantly increasing the amount of available data in this vulnerable population. A significant correlation between daily dose and RIS_am_ concentration was found with a high inter individual variability. As patients with EPS had higher risperidone concentrations than patients without, TDM is recommended to prevent ADRs. Our data hint on a lower therapeutic concentration range in children and adolescents compared to the therapeutic window established for adults. Considering individual pharmacokinetic parameters, TDM provides an effective pharmacovigilance tool in the pediatric population.
